# Coronary Tortuosity Index vs. Angle Measurement Method for the Quantification of the Tortuosity of Coronary Arteries in Non-Obstructive Coronary Disease

**DOI:** 10.3390/diagnostics14010035

**Published:** 2023-12-23

**Authors:** Petra Zebic Mihic, Jerko Arambasic, Drazen Mlinarevic, Sandra Saric, Marina Labor, Ivica Bosnjak, Ivica Mihaljevic, Ines Bilic Curcic, Iva Juric

**Affiliations:** 1 Department of Cardiovascular Diseases, University Hospital Center Osijek, 31000 Osijek, Croatia; zebicmihic@gmail.com (P.Z.M.); iva.os21@gmail.com (I.J.); 2 Faculty of Medicine Osijek, J.J. Strossmayer University of Osijek, 31000 Osijek, Croatia; 3 Cancer and Lung Health Care Unit, University Hospital Linköping, 58185 Linköping, Sweden; 4 Department of Nuclear Medicine, University Hospital Center Osijek, 31000 Osijek, Croatia; 5 Department of Endocrinology, University Hospital Center Osijek, 31000 Osijek, Croatia

**Keywords:** coronary tortuosity, non-obstructive coronary artery disease, myocardial ischemia, tortuosity index, tortuosity angle measurement

## Abstract

Coronary tortuosity has been recognized as a potential pathophysiological mechanism in the development of non-obstructive coronary artery disease (CAD). The aim of this study was to examine the role of two coronary tortuosity measurement methods in the detection of clinically significant coronary tortuosity. The study included 160 patients with angina symptoms and myocardial ischemia detected by cardiac stress tests in chronic settings and those diagnosed with acute coronary syndrome. After coronary angiography, tortuosity of coronary arteries was assessed by two methods, including measurement of tortuosity angles and calculating of tortuosity index. Significantly more tortuous coronary arteries were detected in the group with non-obstructive CAD (*p* < 0.01 for all three arteries), with significantly higher tortuosity index (TI) for all three coronary arteries in this group of patients, compared to patients with obstructive CAD. The highest TI for LCX was found in patients with lateral ischemia (*p* < 0.001) and for LAD in patients with anterior ischemia (*p* < 0.001). When measured by the angle method, the only association was found between LCX tortuosity and lateral ischemia (OR 4.9, *p* = 0.046). In conclusion, coronary tortuosity represents a pathophysiological mechanism for myocardial ischemia in non-obstructive CAD. The coronary tortuosity index could be a reliable and widely applicable tool for the quantification of coronary tortuosity.

## 1. Introduction

Ischemic heart disease represents a significant global health challenge and stands as one of the leading causes of mortality and disability [1]. Traditionally, obstructive coronary artery disease is considered as the main pathophysiologic substrate for the development of myocardial ischemia. It has been defined as the presence of at least 50% reduction in luminal diameter observed during coronary angiography, which subsequently guides therapeutic decisions [2].

However, a substantial proportion of patients, up to 70%, who undergo coronary angiography due to angina and evidence of myocardial ischemia do not exhibit significant narrowing of coronary arteries [2]. Nevertheless, these patients manifest symptomatic ischemia [3]. In recent years, this clinical scenario has gained increasing attention, leading to the adoption of the term “Ischemia with Non-Obstructive Coronary Arteries” (INOCA) to characterize these individuals. Contrary to prior beliefs, accumulating evidence suggests that patients with INOCA are at increased risk for future major adverse cardiac events, including mortality, nonfatal myocardial infarction, nonfatal stroke, and hospitalization for heart failure or angina [4,5]. Furthermore, it was shown that individuals diagnosed with INOCA have a reduced quality of life, increased physical limitations, and a higher frequency of angina when compared to those with stable coronary artery disease [6]. A certain proportion of patients also experience a myocardial infarction with angiography finding of non-obstructive coronary arteries (stenosis < 50% in any major epicardial vessel), with prevalence of up to 14% of patients with acute coronary syndrome [7]. 

Coronary tortuosity, an angiographic finding which is often recognized but inadequately documented by cardiologists, has emerged as a potential contributor to non-obstructive coronary artery disease (CAD). The underlying pathophysiological mechanism likely involves alterations in coronary blood flow, including shear stress-induced friction and the centrifugal effect within curved segments, leading to decreased distal perfusion pressure and the potential onset of myocardial ischemia [8,9]. Hence, shifting the focus from coronary obstruction to other causes of coronary circulation deficiency has become crucial for further understanding of ischemic heart disease.

Tortuosity may occur in practically every artery of any size, such as retinal, vertebral, carotid, coronary, iliac, or the aorta [10]. Quantification of tortuosity is widely used for different arteries but rarely for coronary arteries. This could be due to the complexity of coronary circulation, which is affected by the cyclic systolic and diastolic movements of the heart that make this measurement more complicated. But exactly this dynamic pattern of the heart function with addition of hypertrophic and geometrical changes of the heart caused by chronic pressure or volume overload makes the coronary arteries more prone to tortuosity than other vessels [11]. This explains the association of coronary tortuosity with advanced age, hypertension, and impaired left ventricular relaxation [8,12]. Aging affects coronary circulation through several mechanisms, such as increased stiffness of arteries and myocardium, altered blood pressure control, and higher levels of oxidative stress and inflammation [13]. Female gender is also a risk factor for coronary tortuosity, while the influence of atherosclerosis remains controversial [8,14].

The definition of coronary tortuosity varies across the literature and is often subject to individual interpretation [8,15,16,17]. Several methods for quantifying coronary tortuosity have been proposed in the literature, including the calculation of the tortuosity index or assessment of the number and degree of curvatures. However, there is no generally accepted definition or established severity gradation of coronary tortuosity. Therefore, some differences between earlier studies regarding the extent of coronary tortuosity exist. A widely used definition of coronary tortuosity is the presence of three or more consecutive curvatures (defined as ≥45° change in vessel direction) along the main trunk of at least one coronary artery (left anterior descending (LAD), left circumflex (LCX), and right coronary artery (RCA)) present in both systole and diastole, which some authors classify as severe tortuosity [12,14,16,18,19,20]. Elaid et al. used a more stringent definition, considering severe coronary tortuosity if there are ≥2 consecutive curvatures of ≥180° in a major epicardial coronary artery ≥2 mm in diameter measured at end-diastole [15]. Estrada et al. defined severe coronary tortuosity as the presence of at least three consecutive bends with an angle of less than 90 degrees of an epicardial coronary artery greater than 2 mm during diastole [8]. Zegers et al., determined coronary tortuosity as two or more segments of the coronary arteries with ≥3 curvatures ≤120° during diastole [9]. Notably, there is a scarcity of comprehensive studies quantifying the coronary tortuosity index, which was rarely used as a tool for the gradation of coronary tortuosity [11,21]. 

Establishing a uniform, simple, and reproducible method for assessment of the presence and severity of coronary tortuosity is of paramount importance. The aim of our study was to examine the value of tortuosity index measurement in the assessment of the severity of coronary tortuosity compared to previously used methods of determining the number and degree of curvatures. We also analyzed the association of coronary tortuosity of each epicardial coronary artery with the region of ischemia found during noninvasive diagnostic procedures in order to demonstrate the direct impact of coronary tortuosity on the development of myocardial ischemia.

## 2. Materials and Methods

### 2.1. Patients and Procedures

**Figure 1:**
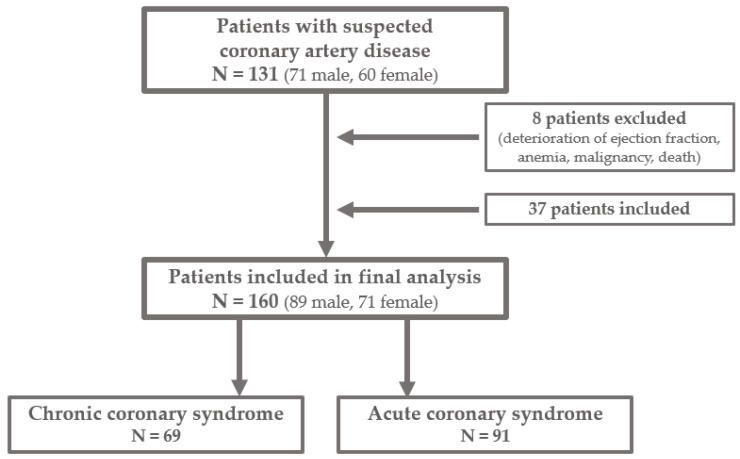
Flow chart of study population selection.

This study represents the additional analysis of the extended sample of patients from our previously conducted study [14]. The study was designed as single-center cross-sectional research which involved 160 participants recruited from December 2021 to July 2023 at the University Hospital Center Osijek. The selection of patients is presented in Figure 1. Additional analysis was conducted in order to examine the utility of rarely used tortuosity index measurement in the detection of non-obstructive CAD and to investigate the association of coronary tortuosity and ischemic alterations detected in different myocardial regions, which would contribute to better understanding of the non-obstructive CAD pathophysiology. Analysis included measurement of coronary tortuosity index on previously obtained coronary angiograms and detailed analysis of stress tests and echocardiograms to assess the exact regions of myocardial ischemia for comparison with the extent of coronary tortuosity of main epicardial arteries measured by both methods, tortuosity index, and the number and degree of bend angles. Approval was obtained from the Institutional Ethics Committee, and all patients provided written consent before participation.

The study included 89 men and 71 women, aged 18 and older, with clinical indications for invasive coronary angiography due to acute coronary syndrome (ACS) or chronic coronary syndrome (CCS). ACS patients presented with unstable angina, non-ST elevation myocardial infarction (NSTEMI), or ST-elevation myocardial infarction (STEMI), while CCS patients exhibited stable angina symptoms with documented coronary ischemia on exercise ECG stress testing or myocardial perfusion scintigraphy. All patients were treated according to current guidelines for the management of acute and chronic coronary syndromes of the European Society of Cardiology [3,7]. After diagnostic coronary angiography, revascularization was performed according to guidelines and clinical judgement of a cardiologist [3,7]. All patients were treated with antiplatelet therapy (aspirin and P2Y12 inhibitors), anticoagulant therapy (unfractionated heparin, low-molecular-weight heparin, or fondaparinux), anti-ischemic drugs (beta-blockers and/or calcium channel blockers, nitrates, and trimetazidine), statins and, depending on comorbidities and cardiovascular risk, angiotensin-converting enzyme (ACE) inhibitors or angiotensin-receptor blockers (ARBs), additional lipid-lowering therapy, antidiabetic, and other specific drugs. We gathered demographic and medical data from all patients, including gender, age, family medical history, and concurrent conditions such as arterial hypertension, diabetes mellitus, and hyperlipidemia, as well as information on smoking and alcohol use, as previously outlined in our publication [14]. All patients provided laboratory findings of complete blood count, lipid profile, electrolytes, markers of renal and liver function, and C-reactive protein. Laboratory tests of complete blood count for all patients were analyzed in order to exclude anemia, while other laboratory findings were not the subject of this study. 

Coronary angiography, via radial or femoral access, was performed using the Philips Azurion 7 M20 radiography unit (Philips Healthcare, Eindhoven, Netherlands) at 15 frames per second. Offline analysis was conducted by two blinded interventional cardiologists. Freeze frames from cine loops obtained during invasive coronary angiography were used for the analysis of the left anterior descending (LAD), left circumflex (LCX), and right coronary artery (RCA). Assessment of coronary arteries was performed from two-dimensional angiograms. The left anterior descending was analyzed in antero-posterior (AP) cranial or right anterior oblique (RAO) cranial views. The left circumflex was assessed in AP caudal or left anterior oblique (LAO) caudal views. The right coronary artery length was analyzed in LAO and straight AP views. All three coronary arteries were measured in the views with the least foreshortening, which differ in each patient due to anatomical factors. 

**Figure 2:**
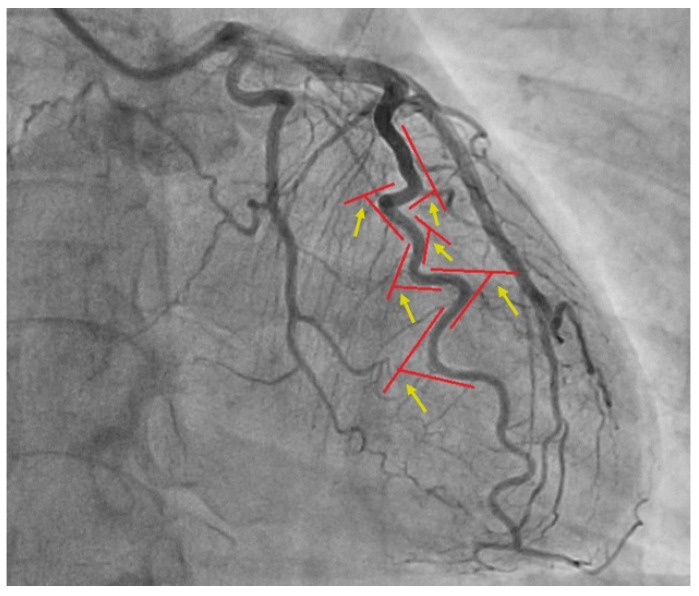
Coronary angiogram demonstrating the angle measurement. Legend: Red lines are representing the direction of coronary vessel; yellow arrows are representing the angle of tortuosity.

Patients were classified as having obstructive coronary artery disease when luminal obstruction of ≥50% was measured in at least one major coronary artery on angiography, while those with obstruction less than 50% were diagnosed with non-obstructive CAD [7]. We used the current definition of non-obstructive coronary artery disease recommended in latest guidelines of the European Society of Cardiology [7]. Coronary tortuosity was assessed by two different methods. The first method included evaluation of the number and degree of coronary artery curvatures, defining coronary tortuosity as the presence of ≥3 consecutive bends, equivalent to a ≥45° change in vessel direction, in at least one major coronary artery during both systole and diastole (Figure 2). 

**Figure 3:**
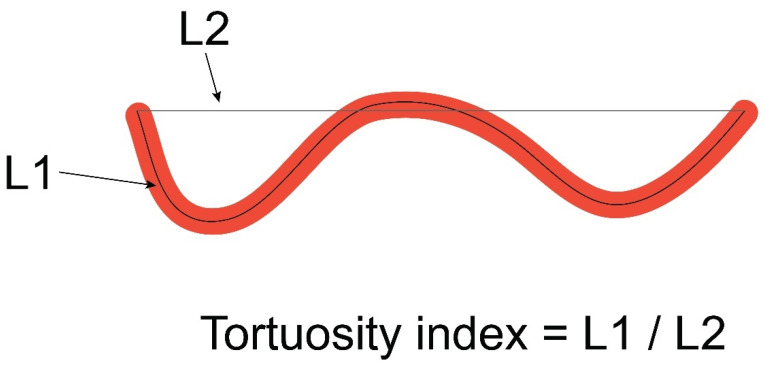
Graphical representation of the tortuosity index measurement. Legend: L1—absolute length of the coronary artery; L2—straight-line length of the coronary artery.

The second method consisted of the assessment of coronary tortuosity index, defined as the ratio between the absolute length of the coronary artery and straight-line length (Figure 3).

**Figure 4:**
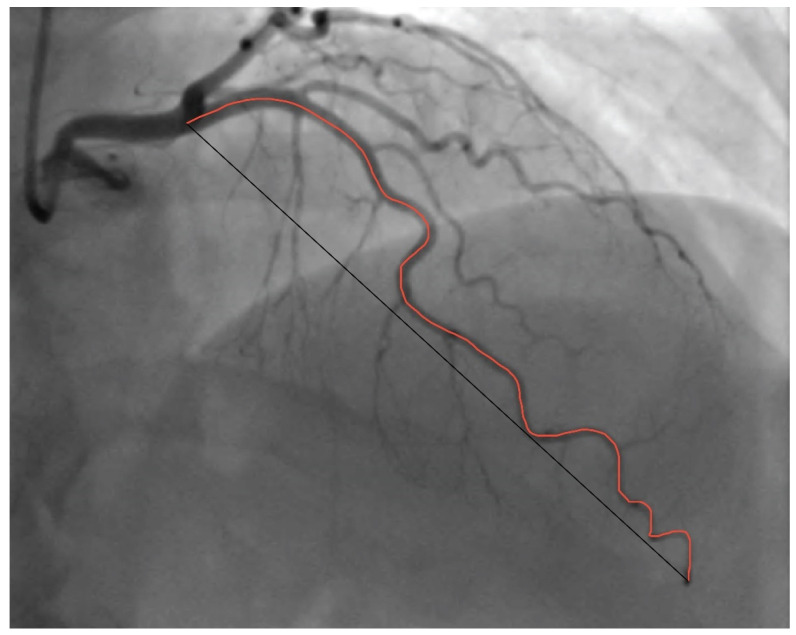
Coronary angiogram demonstrating the tortuosity index measurement. Legend: Red line is representing absolute length of the coronary artery; black line is representing straight-line length of the coronary artery.

Absolute vessel length was measured in diastole from the ostium to the smallest branch visible on angiography and, afterwards, the straight-line length between these two points was measured as well (Figure 4). The analysis was performed using ImageJ software v.154f [22]. After acquiring the freeze-frame image from coronary angiography cine loops, the arteries were measured using the segmented line tool, providing the length in pixels. 

In subjects with non-obstructive CAD, we examined the correlation between coronary artery tortuosity and ischemic changes in the associated myocardial area. In patients with non-obstructive CAD who presented as chronic coronary syndrome, ischemic changes were evaluated by exercise ECG stress testing in 34 patients and by myocardial perfusion scintigraphy in 14 patients. Although there are several proposed noninvasive imaging techniques, we used exercise ECG stress test as well established and the most accessible stress modality which is recommended by the European Society of Cardiology and American Heart Association as an option in diagnostic evaluation of chest pain to clarify the clinical probability of the presence of obstructive CAD [3,23]. 

We categorized participants into three groups, representing myocardial ischemia in specific regions: anterior, lateral, and inferior. During exercise stress testing, myocardial ischemia was detected with regard to the ECG changes associated with the specific myocardial supply region. The supply areas were defined as follows: LAD—septal and anterior ischemia included leads V1–V4; LCX—lateral ischemia included leads I, aVL, V5, and V6; and RCA—inferior ischemia included leads II, III, and aVF [24]. Myocardial perfusion scintigraphy revealed ischemia according to the myocardial regions with reduced absorption of radiopharmaceutical. In patients with acute coronary syndrome and non-obstructive CAD, we evaluated ischemic changes observed by echocardiography, which were found in 23 patients, while 14 patients did not have echocardiographic signs of ischemia. Areas of myocardium included 15 segments divided by the supplying coronary artery: LAD—septal and anterior ischemia is associated with segments 1, 2, 6, 7, 11 and 12; LCX—lateral ischemia is associated with segments 9 and 10; and RCA—inferior ischemia is associated with segments 4, 5, 14, and 15. Apical segments 3, 8, and 13 are irrigated by all three coronary vessels [25]. 

All patients underwent standardized transthoracic two-dimensional echocardiography performed using a GE Vivid E9 device (GE HealthCare, Chicago, IL, USA). Wall motion abnormalities were identified by the 17-segment model recommended by the American Society of Echocardiography. According to the regions of wall motion abnormalities, LAD is associated with anterior septum and anterior free wall, LCX with lateral regions, and RCA with inferior septum and inferior free wall [26,27].

Exclusion criteria included left ventricular systolic dysfunction with ejection fraction < 50%, diastolic dysfunction beyond grade I, pulmonary hypertension, congenital and valvular heart diseases, myocardial bridging, anemia, malignancies, chronic obstructive pulmonary disease, and musculoskeletal and psychological disorders.

### 2.2. Statistical Analysis 

Statistical analysis was performed using the statistical software packages Statistica v.12 (StatSoft, Inc., Tulsa, OK, USA) and MedCalc v.20 (MedCalc Software Ltd., Ostend, Belgium). Categorical variables were presented as numbers and percentage (%) and, for their comparisons between groups, chi-square test was used. Continuous variables were presented as mean with standard deviations or medians and interquartile range (IQR) depending on the normality of distribution. Comparisons between groups were conducted using Student’s *t*-test or Mann–Whitney depending on the distribution or, for multigroup comparisons, using Kruskal–Wallis test. The association of variables (continuous and categorical) was determined using univariate logistic regression analysis. *p* < 0.05 was used as statistically significant for all performed analyses.

## 3. Results

The study enrolled 160 patients, of whom 55.6% were men and 44.4% women. After the coronary angiography, according to the findings, 85 patients were classified as non-obstructive CAD (NOCAD) and 75 as obstructive CAD (OCAD). Their demographics and personal history data are presented in Table 1.

The groups were comparable for age (61 vs. 62 years, *p* = 0.490) and BMI (27.75 vs. 28.40 kgm^−2^, *p* = 0.285). There were significantly more women (60.0% vs. 26.7%, χ^2^ = 17.8, *p* < 0.001) and non-smokers (75.3% vs. 56.0%, χ^2^ = 6.6, *p* = 0.010) in the NOCAD group. Hypertension, hyperlipidemia, and positive family history of cardiovascular disease were present in comparable proportions (*p* > 0.05 for all), while diabetes was more prevalent in OCAD group (34.7% vs. 20.0%, χ^2^ = 4.3, *p* = 0.037). Considering the diagnosis of referral, significantly more patients presented with chronic coronary syndrome (CCS) in the NOCAD group than in the OCAD group (56.5% vs. 28.0%, χ^2^ = 13.1, *p* < 0.001, Table 1). 

As seen in Table 2, there were significantly more tortuous coronary arteries found in NOCAD compared to OCAD (*p* < 0.01 for all three arteries), measured by angle method. When the tortuosity index (TI) of coronary arteries were compared, TI was higher in NOCAD compared to OCAD patients with a significant difference for all three coronary arteries (median LCX 1.30 vs. 1.15, *p* < 0.001; median LAD 1.26 vs. 1.18, *p* < 0.001; median RCA 2.00 vs. 1.87, *p* = 0.014; Table 2). 

A significant association between the presence of angle-measured tortuosity for LCX and TI for LCX was found (logistic regression coefficient ± SE, 13.4 ± 2.2, *p* < 0.001) as well as between angle-measured tortuosity for LAD and TI for LAD (logistic regression coefficient ± SE, 14.6 ± 2.4, *p* < 0.001) but not for RCA (*p* = 0.506, logistic regression). 

**Figure 5:**
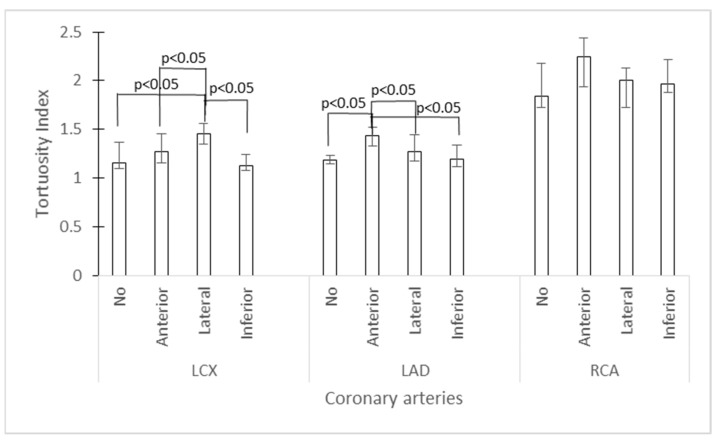
Graphical representation of association of the tortuosity index by coronary artery with regions of myocardial ischemia. Legend: LCX—left circumflex artery; LAD—left anterior descending artery; RCA—right coronary artery); bars represent median values and whiskers represent the interquartile range (Kruskal–Wallis test).

There was a significant association of TI for LCX and LAD with specific regions of myocardial ischemia. The highest TI for LCX was found in patients with lateral ischemia (median 1.45, IQR 1.35–1.56; *p* < 0.001; Kruskal–Wallis test; post hoc significantly different to anterior and inferior ischemia or no ischemia) and for LAD in patients with anterior ischemia (median 1.43, IQR 1.33–1.52; *p* < 0.001; Kruskal–Wallis test; post hoc significantly different to lateral and inferior ischemia or no ischemia). An association of TI for RCA and inferior ischemia was not found (*p* = 0.122) (Figure 5). 

When measured by angle method, a significant association between LCX tortuosity and lateral ischemia was observed (OR 4.9, 95% CI 1.0 to 23.6, *p* = 0.046, logistic regression). For tortuosity of LAD, the association did not reach significance but it was associated with anterior ischemia (OR 0.2, for lateral ischemia and OR < 0.1 for inferior ischemia, logistic regression). There was no significant association of RCA tortuosity with myocardial ischemia (*p* = 0.222, logistic regression).

**Figure 6:**
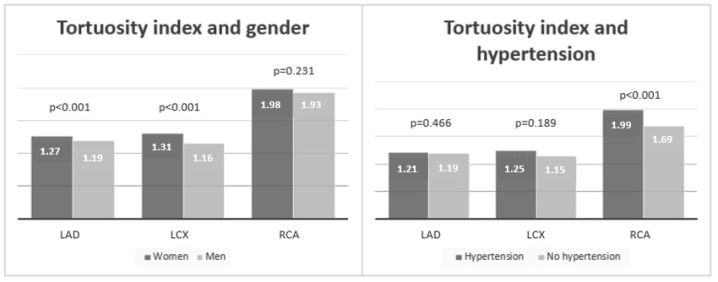
Graphical representation of association of the tortuosity index by coronary artery with gender and hypertension. Legend: LCX—left circumflex artery; LAD—left anterior descending artery; RCA—right coronary artery (RCA); bars represent median values of tortuosity index.

We found a significantly higher TI of LCX and LAD in women (median LCX 1.31 vs. 1.16, *p* < 0.001; median LAD 1.27 vs. 1.19, *p* < 0.001) but not of RCA (median RCA 1.98 vs. 1.93, *p* = 0.231) (Figure 6). On the other hand, for patients with arterial hypertension, a significant difference for TI was found only for RCA (median RCA 1.99 vs. 1.69, *p* < 0.001) but not for LCX and LAD (median LCX 1.25 vs. 1.15, *p* = 0.189; median LAD 1.21 vs. 1.19, *p* = 0.466) (Figure 6). Regarding age, there was a mild but significant association found only for TI of RCA (R = 0.282, *p* < 0.001) but not for TIs of LCX (R = 0.03, *p* = 0.696) and LAD (R = 0.06, *p* = 0.452).

## 4. Discussion

Frequently noticed at coronary angiography but never truly seen and measured, coronary tortuosity deserves more attention in everyday clinical practice. There is a significant proportion of patients with signs and symptoms of myocardial ischemia but without obstruction of coronary arteries. Detection of the exact pathophysiological substrate of non-obstructive CAD would improve diagnostic and therapeutic strategies for these patients.

In our study, more than 50% of patients were classified as non-obstructive CAD after coronary angiography, while, in the group of patients with chronic symptoms and ischemic alterations who came for elective coronary angiography, almost 70% presented as non-obstructive CAD. This finding is consistent with previous studies which showed that up to 70% of patients with angina symptoms and myocardial ischemia do not have significant obstructions of coronary arteries [2,8]. Such a high incidence of angiographic findings with non-obstructive coronary arteries in patients with angina symptoms and myocardial ischemia highlights that there is a significant coronary circulation pathology beyond obstructive disease.

Several previous studies showed that coronary tortuosity may have an important role in the development of ischemic alterations in non-obstructive coronary artery disease [8,14,28]. Our study confirms this finding since we detected significantly more tortuous coronary arteries in the group of patients without significant obstruction of coronary arteries, including all three main coronary arteries. This finding suggests that coronary tortuosity may play a role in the pathophysiology of non-obstructive CAD. As previously discussed, a possible mechanism for ischemia is the reduction in perfusion pressure and coronary flow distal to the tortuous segments of arteries due to friction and centrifugal effect [8,9,15]. Sharp angles of the tortuous artery disrupt normal laminar flow, causing significant energy loss and insufficient perfusion pressure downstream [15].

However, there is no generally accepted definition or established severity gradation of coronary tortuosity. Previous studies used various methods for quantification, making interpretation and comparison of the results difficult [8,9,11,15,17,21]. In our study, we used two methods of tortuosity measurement and examined the difference and their association with non-obstructive CAD and myocardial ischemia [11,14,21]. 

When measured by angle method, there were significantly more tortuous coronary arteries in the group of patients with non-obstructive CAD compared than those with obstructive CAD, even when compared by each artery individually. Measurement of the tortuosity index also showed a significantly higher tortuosity in the group of patients with non-obstructive CAD in comparison with the group with obstructive CAD, with significant differences observed for all three coronary arteries. Among the patients with non-obstructive CAD, almost 60% had coronary tortuosity, indicating the potential causative association of these two entities. In the group of patients with non-obstructive CAD, most had a tortuous LCX, followed by LAD and significantly less RCA. This finding is consistent with previous studies [14,15,18,29]. This difference between coronary arteries according to their predisposition for tortuosity is not yet clarified. It is proposed that it could be associated with myocardial regional thickness differences [14], but this marked non-tortuosity of RCA could be a result of the specific curved shape of this artery. Only 5% of our patients showed tortuosity of RCA and all of them were in the group with non-obstructive CAD. Non-tortuous RCA could be a direct consequence of the natural RCA shape, mildly curved in its whole course, which makes them less prone to the development of tortuosity. It is known that C-shaped RCA is associated with atherosclerosis with most narrowings located in the proximal region due to hemodynamic characteristics [30], while we noticed that most tortuosity of RCA is located in distal parts of the vessel. Bends of tortuous coronary arteries change the hemodynamic pattern in a way that protects them from atherosclerosis, which explains the significantly less tortuous coronary arteries in obstructive CAD. This observation was consistent with some previous studies [14,31,32,33], although there are also some opposing conclusions [34,35].

When comparing the tortuosity of each coronary artery measured by angle method, with myocardial ischemia of the corresponding region, there was a significant correlation between the tortuosity of LCX and ischemia of the lateral region of myocardium supplied by LCX. Tortuosity of LAD showed association with the anterior ischemia but the result did not reach significance. There was no significant relationship between RCA tortuosity and myocardial ischemia. Estrada et al. also found more pronounced tortuosity in the LCX and the greatest frequency of ischemia in that region [8]. The association of tortuosity index and myocardial ischemia of the specific regions showed a significant level for LCX and LAD but not for RCA. The tortuosity index of LCX was significantly higher in the group of patients with lateral ischemia, while the tortuosity index of LAD was significantly associated with anterior ischemia. An association between the tortuosity index of RCA and myocardial ischemia was not found. The absence of connection between RCA tortuosity and ischemic changes of the associated myocardial area could be due to a small sample or because of previously mentioned tortuosity of more distal parts of RCA that do not cause such a significant pressure reduction to induce evident ischemia.

Female gender, hypertension, and advanced age are found to be significant risk factors for tortuosity in previous studies [8,12,19]. Our study showed significantly higher TI for CX and LAD in women, while TI index for RCA did not reach statistical significance. The cause of this relationship between female gender and tortuosity is not yet clarified. Regarding arterial hypertension and age, a significant difference for TI was found only for RCA but not for LCX and LAD. Since RCA is anatomically less prone to tortuosity, the contribution of hypertension and aging in the development of tortuosity of this vessel has a more significant role.

Although we used a less stringent definition of the severe coronary tortuosity measured by the angle method, we showed that this extent of tortuosity is severe enough to cause significant myocardial ischemia. Since we showed a direct association of coronary tortuosity and myocardial ischemia of specific supply regions, the role of tortuosity in the development of non-obstructive CAD seems undeniable. Regarding the difference between two methods for measurement, we found a significant association between the presence of angle-measured tortuosity and TI for LCX and LAD, showing that those methods correlate well. The tortuosity index method showed a significant relationship of LCX tortuosity with lateral ischemia and LAD tortuosity with anterior ischemia. The angle measurement method showed a significant association only for LCX; thus, it could be concluded that the tortuosity index method is more reliable for the detection of tortuosity and quantification of severity. Marking the whole length of the coronary artery makes this method more time-consuming, but it is definitely a more precise method in detecting myocardial ischemia. Tortuosity index gives a concrete quantification of tortuosity, while the angle method often relies on visual assessment. Both methods showed a significant relationship of tortuosity and non-obstructive CAD; however, the angle measurement method is good for quick visual detection of significant tortuosity as opposed to the index measurement method, which could be a great tool for accurate quantification of severity.

Our study brings new evidence that confirms the direct impact of coronary tortuosity on the development of myocardial ischemia as one of the important contributors in the development of non-obstructive CAD. We examined the feasibility of available tools for the quantification of coronary tortuosity to determine the most accurate method for implementation in clinical practice. This is, to our knowledge, the first study evaluating coronary tortuosity index in the clinical setting of myocardial ischemia. 

This study also has some limitations. Although the number of patients included is sufficient to achieve valuable and statistically significant results, the sample is too small for establishing the reference values for the tortuosity index. Uni-centricity of the study is another limitation. In order to set the reference values for the tortuosity index, it is necessary to recruit a much larger group of patients from multiple centers in future studies. Using the exercise ECG stress test as a diagnostic procedure in evaluation of patients with suspected CAD is also a limitation, considering that there are more reliable functional noninvasive tests which should be used in future research. 

Future studies should be directed to establish the reference values for measurement of tortuosity. Development of computer software that would mark the length of coronary arteries from freeze-frame coronary angiography images without the need for manual measurement would lead to easier implementation of tortuosity measurement in clinical practice. Furthermore, considering that microvascular dysfunction is an important contributor in the development of myocardial ischemia in non-obstructive CAD, further investigation of etiology and potential multifactorial cause of this disease is needed.

Since this clinical entity is recognized as an important contributor to the development of myocardial ischemia, there is a need to implement the measurement of coronary tortuosity to routine coronary angiography analysis, especially in patients without significant obstruction of coronary arteries. In order to achieve that, some simple, uniform, widely applicable, and reproducible method of measurement should be established. Development of an accessible and reliable tool for quantification of the extent of coronary tortuosity on two-dimensional coronary angiography recordings would lead to earlier recognition and diagnosis of the non-obstructive CAD caused by ischemic alterations due to increased tortuosity of coronary arteries. Patients with non-obstructive CAD are found to be at increased risk for major cardiovascular events, including cardiovascular death and all-cause mortality [36]. Considering that these are underdiagnosed high-risk patients, future research should include the determination of an appropriate individualized cardiovascular risk assessment tool for these patients beyond existing risk scores, which would include underutilized noninvasive cardiovascular imaging techniques [37]. 

## 5. Conclusions

Coronary tortuosity represents a pathophysiological mechanism for the development of myocardial ischemia in non-obstructive CAD. The tortuosity index of coronary arteries is a more accurate method than angle measurement and could be a reliable and widely applicable tool for quantification of coronary tortuosity. 

## Figures and Tables

**Table 1:** Demographics and personal history data of the study population divided into 2 groups according to the presence of coronary artery obstruction (*N* = 160).

	Group		
Variable	NOCAD (*N* = 85)	OCAD (*N* = 75)	Statistics
Female; *N* (%)	51 (60.0)	20 (26.7)	χ^2^ = 17.8, *p* < 0.001
Age, yrs; mean (SD)	61.0 (10.7)	62.1 (9.5)	0.490 *
BMI, kgm^−2^; M (IQR)	27.75 (25.92–31.36)	28.40 (26.24–31.33)	0.285 **
Smoking; N (%)			χ^2^ = 6.6, *p* = 0.010
Non smokers	64 (75.3)	42 (56.0)	
Smokers	21 (24.7)	33 (44.0)	
*Medical history*; *N* (*%*)			
DM	17 (20.0)	26 (34.7)	χ^2^ = 4.3, *p* = 0.037
HTA	68 (80.0)	56 (74.7)	χ^2^ = 0.6, *p* = 0.422
HLP	38 (44.7)	42 (56.0)	χ^2^ = 2.0, *p* = 0.155
*Diagnosis of referral*; *N* (*%*)			χ^2^ = 13.1, *p* < 0.001
CCS	48 (56.5)	21 (28.0)	
ACS	37 (43.5)	54 (72.0)	

Legend: * Student’s *t*-test; ** Mann–Whitney U-test; SD—standard deviation, M—median, IQR—interquartile range, χ^2^—chi-square, NOCAD—non-obstructive coronary artery disease, OCAD—obstructive coronary artery disease, BMI—body mass index, DM—diabetes, HTA—arterial hypertension, HLP—hyperlipidemia, CCS—chronic coronary syndrome, ACS—acute coronary syndrome.

**Table 2:** Tortuosity of coronary arteries and type of blood supply according to groups (*N* = 160).

	Group		
Variable	NOCAD (*N* = 85)	OCAD (*N* = 75)	Statistics
Tortuous coronary artery; *N* (%)			
LCX	34 (40.0)	11 (14.7)	χ^2^ = 12.6, *p* < 0.001
LAD	28 (32.9)	8 (10.7)	χ^2^ = 11.3, *p* < 0.001
RCA	8 (9.4)	0 (0.0)	χ^2^ = 7.38, *p* = 0.007
Tortuosity index; M (IQR)			
LCX	1.30 (1.13–1.47)	1.15 (1.10–1.30)	*p* < 0.001 *
LAD	1.26 (1.18–1.45)	1.18 (1.14–1.26)	*p* < 0.001 *
RCA	2.00 (1.79–2.27)	1.87 (1.65–2.17)	*p* = 0.014 *
Myocardial ischemia; *N* (%)			
Anterior	20 (23.5)		
Lateral	35 (41.2)		
Inferior	16 (18.8)		
None	14 (16.5)		

Legend: * Mann–Whitney U-test; M—median, IQR—interquartile range, χ^2^—chi-square, NOCAD—non-obstructive coronary artery disease, OCAD—obstructive coronary artery disease LCX—left circumflex coronary artery, LAD—left anterior descending coronary artery, RCA—right coronary artery.

## Data Availability

The data presented in this study are available on request from the corresponding author. The data are not publicly available due to ethical reasons.
